# Roles of thermophilic thiosulfate-reducing bacteria and methanogenic archaea in the biocorrosion of oil pipelines

**DOI:** 10.3389/fmicb.2014.00089

**Published:** 2014-03-06

**Authors:** Renxing Liang, Robert S. Grizzle, Kathleen E. Duncan, Michael J. McInerney, Joseph M. Suflita

**Affiliations:** Department of Microbiology and Plant Biology, OU Biocorrosion Center, University of OklahomaNorman, OK, USA

**Keywords:** thiosulfate reducing bacteria, sulfidogenesis, methanogenesis, *Anaerobaculum*, *Methanothermobacter*, biocorrosion

## Abstract

Thermophilic sulfide-producing microorganisms from an oil pipeline network were enumerated with different sulfur oxyanions as electron acceptors at 55°C. Most-probable number (MPN) analysis showed that thiosulfate-reducing bacteria were the most numerous sulfidogenic microorganisms in pipeline inspection gauge (PIG) scrapings. Thiosulfate-reducing and methanogenic enrichments were obtained from the MPN cultures that were able to use yeast extract as the electron donor. Molecular analysis revealed that both enrichments harbored the same dominant bacterium, which belonged to the genus *Anaerobaculum.* The dominant archaeon in the methanogenic enrichment was affiliated with the genus *Methanothermobacter*. With yeast extract as the electron donor, the general corrosion rate by the thiosulfate-reducing enrichment (8.43 ± 1.40 milli-inch per year, abbreviated as mpy) was about 5.5 times greater than the abiotic control (1.49 ± 0.15 mpy), while the comparable measures for the methanogenic culture were 2.03 ± 0.49 mpy and 0.62 ± 0.07 mpy, respectively. Total iron analysis in the cultures largely accounted for the mass loss of iron measured in the weight loss determinations. Profilometry analysis of polished steel coupons incubated in the presence of the thiosulfate-reducing enrichment revealed 59 pits over an area of 71.16 mm^2^, while only 6 pits were evident in the corresponding methanogenic incubations. The results show the importance of thiosulfate-utilizing, sulfide-producing fermentative bacteria such as *Anaerobaculum* sp. in the corrosion of carbon steel, but also suggest that *Anaerobaculum* sp. are of far less concern when growing syntrophically with methanogens.

## Introduction

The United States alone has about 2.5 million miles of pipelines that crisscross the country and transport the majority of energy reserves (Quickel and Beavers, 2011). Corrosion is a major cause of pipeline failures that can lead to economic loss and severe environmental contamination (Kilbane and Lamb, 2005). It is well recognized that the majority of internal corrosion of oil pipelines is associated with microorganisms (Almahamedh et al., 2011). Previous studies using culture-independent approaches demonstrated that various physiological groups of microbes like sulfate-reducing bacteria, fermentative bacteria, metal reducers and methanogens are the frequent and near ubiquitous inhabitants in pipelines transporting hydrocarbon reserves (Neria-González et al., 2006; Duncan et al., 2009; Keasler et al., 2010; Rajasekar et al., 2010; Stevenson et al., 2011). It was hypothesized that the resident microorganisms inside the pipelines contribute to corrosion under the prevailing anaerobic conditions through their metabolic ability to use a wide variety of electron acceptors such as sulfate and thiosulfate (Suflita et al., 2008).

The production of corrosive sulfides by sulfidogenic organisms is an important mechanism of pipeline corrosion (Davidova et al., 2012). Sulfate-reducing bacteria are considered to be the major corrosion culprits and have been most extensively studied (Hamilton, 1985; Beech and Sunner, 2004). However, previous work suggested that strict sulfate-reducing bacteria may be present in lower numbers than other sulfide producers including organisms that reduce thiosulfate (Obuekwe et al., 1983; Obuekwe and Westlake, 1987; Crolet, 2005; Agrawal et al., 2010). It was proposed that thiosulfate could be generated by the chemical oxidation of H_2_S with O_2_ (Cline and Richards, 1969). Meanwhile, other studies demonstrated that thiosulfate was produced through electrochemical dissolution of MnS in stainless steel during the initiation of pitting corrosion (Alkire and Lott, 1989; Lott and Alkire, 1989). Indeed, thiosulfate has been detected at concentrations up to 0.5 mM in production facilities (Magot et al., 1997). The biological turnover of thiosulfate can be rapid and potentially a more significant contributor to the sulfide pool when reduced than sulfate alone (Jørgensen, 1990). Therefore, the presence of thiosulfate and its utilization by bacteria can represent a major risk factor for pipeline biocorrosion. In support of this concept, Magot et al. (1997) found that mesophilic, thiosulfate-reducing bacteria from a petroleum pipeline were capable of corroding carbon steel at a rate comparable to the sulfate reducers (Magot et al., 1997).

Previous studies found that sequences related to the genera *Thermoanaerobacter, Anaerobaculum*, and *Thermacetogenium* were dominant in clone libraries and pyrosequencing assays of ANS oil facilities that were operated at approximately 50°C (Duncan et al., 2009; Gieg et al., 2010; Stevenson et al., 2011). Species within these genera are known to be capable of reducing thiosulfate. Although the above thermophilic thiosulfate-reducers were suspected to be involved in corrosion processes, their direct role in sulfide production and biocorrosion was not clearly demonstrated. Moreover, many of these organisms are typically classified as fermentative bacteria and thus, little attention has been given to their role in sulfidogenesis and biocorrosion. It has been noted that anti-corrosion strategies involving biocides that target sulfate-reducing bacteria may be ineffective against thiosulfate-reducing bacteria (Crolet, 2005).

Thermophilic methanogens affiliated with the genus *Methanothermobacter* were also detected at the ANS oil facility (Duncan et al., 2009; Stevenson et al., 2011). It was recognized that the syntrophic association of methanogens with fermentative microorganisms may be important for enhancing corrosion. Co-cultures of *Methanothermobacter* strain MG and *Thermococcus* species produced elevated concentrations of fatty acids through yeast extract fermentation, which can increase the corrosion of carbon steel (Davidova et al., 2012). More importantly, the possibility of direct electron extraction from iron by methanogens has been postulated as an important corrosion mechanism (Dinh et al., 2004; Uchiyama et al., 2010).

Understanding the microbial interactions involved in the cycling of organic substrates and sulfur oxyanions is crucial to the development of strategies to minimize or prevent biocorrosion. We used both cultivation-dependent and -independent techniques to enumerate and identify sulfidogenic and methanogenic microorganisms from a hot ANS petroleum production facility. The potential impact of thiosulfate-reducing bacteria and methanogenic archaea on corrosion of carbon steel was further evaluated.

## Materials and methods

### Sampling source and collection

Samples were collected from a segment of pipeline in the ANS oil production complex that delivered production water from a central processing facility to an injection well (Stevenson et al., 2011). The average temperature of the fluid flowing through this area was approximately 50°C. Samples of the pipeline inspection gauge (PIG) scrapings, which included solid materials scraped from the inner surface of the pipeline and some liquid, were collected in sterile glass serum bottles. The bottles were sealed with rubber stoppers, capped, and flushed with nitrogen to maintain anaerobic conditions (Stevenson et al., 2011). The sample was then shipped to the University of Oklahoma and stored at room temperature under nitrogen gas and used as an inoculum for MPN determinations.

### Media and most-probable number enumerations

A basal marine medium was used to enumerate themophilic sulfide-producing microorganisms capable of using sulfate, sulfite, thiosulfate or polysulfide as electron acceptors (Tanner et al., 2007). The basal marine medium contained the following components (l^−1^): yeast extract, 0.5 g; NaCl, 20.0 g; NaHCO_3_, 1.0 g; CaCl_2_·2H_2_O, 0.04 g; PIPES [piperazine-N,N'-bis(2-ethanesulfonic acid)], 3.786 g (10 mM); a 0.1% resazurin solution, 1 ml; a 2.5% Na_2_S•9H_2_O solution, 20 ml; a modified trace metals solution (Wolin et al., 1963), 10 ml; a modified vitamin solution (Wolin et al., 1963), 0.5 ml; and a marine minerals solution, 10 ml. The marine minerals solution contained (l^−1^): KCl, 200 g; NH_4_Cl, 100 g; MgSO_4_·7H_2_O, 40 g; and KH_2_PO_4_, 20 g. The 2.5% Na_2_S•9H_2_O solution was prepared as described previously (McInerney et al., 1979) and added after boiling and gassing the medium. The pH of the medium was adjusted to 7.0 using 0.1 N HCl or 0.1 N NaOH.

Two sets of basal marine media were prepared to determine the dominant sulfide producers using a most-probable number (MPN) method (McInerney et al., 2007). One set was amended with a mixture of short-chain volatile fatty acids (VFAs) at 200 μM each of formic, acetic, propionic, butyric, and valeric acids as the electron donors. The second set did not have VFAs added. Yeast extract was added to supply additional growth factors and carbon sources likely produced by an active microbial community. Twenty-percent stock solutions of Na_2_SO_4_, Na_2_SO_3_, and Na_2_S_2_O_3_, and a 1 M stock solution of polysulfide (Na_2_S_3.25_) (Widdel and Pfennig, 1992) were added individually at 6 ml·l^−1^ to the basal marine medium with and without VFAs. Media with and without VFAs, both lacking sulfidogenic electron acceptors, were included as controls. The medium was heated, degassed with 100% N_2_, sealed, and brought inside of the anaerobic chamber. The medium was reduced by the addition of the sodium sulfide solution and then dispensed into 16 × 150 mm screw-capped, Hungate tubes, 9 ml per tube. The tubes were sealed with Hungate caps and septa, brought out of the chamber where the gas phase was replaced with an atmosphere of 80% N_2_: 20% CO_2_, as described previously (Tanner et al., 2007), and then autoclaved. Additions, inoculations, and sampling of sterile anoxic media were done using sterile, degassed syringes and needles.

For each condition, each of three tubes was inoculated with 1 ml of PIG envelope sample Subsequent 1:10 serial dilutions were made in triplicate in the same medium as the 1:10 dilution until a final dilution of 10^−6^. All tubes were then incubated at 55°C for 16 weeks. The MPN tubes were scored positive if sulfide production was at least 20% higher than that of the uninoculated and unamended controls. The MPNs tubes containing polysulfide as the electron acceptor were scored positive when the number of cells visible in multiple microscopic fields at 400X was greater (at least two times) than that in control tubes without electron acceptors. The calculation of the MPN was deduced from the statistical table provided previously (Banwart, 1981).

### Characterization of enrichments from MPN cultures

Enrichments for all electron acceptors except polysulfide were established from highest positive MPN dilution tubes. Enrichment media and equipment was the same as that used for the MPN assay, except that rubber stoppers and Balch tubes were used. Two milliliters from a positive MPN tube were transferred into each of three Balch tubes containing 9 ml of the cultivation medium. Controls without VFAs but with an electron acceptor, with VFAs but without an electron acceptor, and lacking both VFAs and a sulfidogenic electron acceptor were prepared in a similar manner. The enrichments were incubated at 55°C for 12 weeks and then transferred to the same fresh medium. After each transfer, samples were taken to measure growth, sulfide production and to obtain DNA for community analysis.

Thiosulfate-reducing and methanogenic enrichments with and without the addition of VFAs were selected for further characterization. Since the enrichment media included yeast extract as well as the VFA solution, the concentration of yeast extract was varied to determine if these enrichments used yeast extract as the electron donor. The thiosulfate-reducing enrichments were grown in medium without VFAs at yeast extract concentrations of 0, 1, 5, and 10 g·l^−1^ with an initial thiosulfate concentration of around 15 mM. The methanogenic enrichment was inoculated into media with the same yeast extract concentrations as above but without thiosulfate. The reduction of thiosulfate, accumulation of sulfide, and methane production were monitored with time.

### Corrosion assay

The carbon steel (1018) used for this study had a composition of 0.14–0.20% C, 0.6–0.9% Mn, 0.035% maximum S, 0.030% maximum P, and the remainder Fe. It was cut into small round coupons with diameters of 9.53 mm and a thickness of approximately 1 mm. The coupons (original weight: 0.5253 ± 0.0077 g) were initially polished to a 3–5 micron finish to form a smooth level surface. The coupons were then cleaned with deionized water, and sonicated for 15 min in distilled water followed by two successive acetone treatments. The coupons were then dried with flush of N_2_, placed in sealed serum bottles under N_2_ and autoclaved.

The coupons were weighed individually and added to the culture tubes aseptically inside an anaerobic chamber. All incubations were performed in triplicate and incubated at 55°C. The protocol for cleaning the coupons at the end of experiment followed the ASTM Standard G1-03 (ASTM G1-03, 2003) with some modification. The acid cleaning solution (3.5 g·l^−1^ of hexamethylene tetramine in 6 M HCl) was prepared to remove accumulated corrosion product from the coupon surface. Ten milliliters of acid cleaning solution were directly added to the cultures with coupons and the cultures were sonicated for 15 min. The coupons were removed and rinsed (in order) with deionized water, acetone, and methanol. The cleaned coupons were dried and stored under N_2_ prior to analysis.

Corrosion was determined by both weight loss and total iron determinations. Individual coupons were weighed in the glove bag as described above. The corresponding general corrosion rate was calculated from mass loss according to NACE Standard RP0775 (RP0775 NACE Standard, 2005). The total iron (including insoluble iron dissolved from the surfaces of coupon and medium) in the acid cleaning solution was reduced with 10% hydroxylamine hydrochloride to reduce any ferric iron to its ferrous state (Stookey, 1970) and then diluted with nanopure water to quantify the total ferrous iron by the ferrozine assay (Lovley and Phillips, 1986).

### Field emission-scanning electron microscopy (FE-SEM) and profilometry

A set of corrosion product-covered coupons were withdrawn from replicate incubations and dried under N_2_. High resolution FE-SEM was performed using a Zeiss NEON 40 EsB (Carl Zeiss, Oberkochen, Germany) scanning electron microscope. Images of the coupon surfaces were obtained using an acceleration voltage of 1 kV and a working distance of 4 mm. Another set of coupons from replicate incubations were cleaned as described above and then scanned with a Nanovea non-contact optical profilometer PS50 (MicroPhotonics, Inc, Irvine, CA) with a 2 μm step size (Harris et al., 2010). The electronically recorded raw data were analyzed using Ultra MountainsMap Topography XT6.0 software (MicroPhotonics, Inc, Irvine, CA). The pits were defined as the regions that were 20 μm below the mean plane and had an equivalent diameter greater than 10 μm. Equivalent diameter is the diameter of an irregular region whose outer circumference equals a circular disk with a diameter of 10 μm.

### Analytical techniques

Dissolved sulfide was measured using a colorimetric assay with N,N-dimethyl-p phenylenediamine·HCl, zinc acetate, and concentrated sulfuric acid (Tanner, 1989). Gas-phase H_2_S concentrations were measured by injecting 1-ml of the enrichment headspace directly into the assay tube containing the above reagent, which was then shaken vigorously prior to conducting the assay. Sulfate was measured by ion chromatography using a Dionex DX500 ion chromatograph equipped with an AS4A column (Caldwell et al., 1998). Thiosulfate concentrations were analyzed with a colorimetric assay (Nor and Tabatabai, 1975) and measured spectrophotometrically at 460 nm. VFAs and methane were analyzed at the beginning and at the end of the incubations by gas chromatography (GC) (Struchtemeyer et al., 2005).

### Community analysis by 16S rRNA gene sequencing

The highest dilution MPN tubes in which all three replicates were scored positive for sulfide production and the resulting enrichments that showed electron acceptor loss and sulfide production above controls were chosen for DNA analyses. Five milliliters of each culture was centrifuged for 15 min at 6000 × g. The cell pellet was washed twice in 1 ml of a sterile phosphate-buffered saline (PBS) followed by centrifugation for 3 min at 8000 × g. The final pellet was resuspended in 500 μl of sterile PBS solution and used immediately for DNA extraction or frozen at −20°C.

DNA was extracted from 250-μl samples using the PowerSoil DNA Isolation Kit (MOBIO Laboratories). Due to low cell biomass, two sets of tubes per sample were pooled onto one spin filter in order to achieve a higher DNA concentration. The DNA was then extracted from the spin filter into 75 μl sterile molecular biology-grade water (Eppendorf, Hamburg, Germany) and stored at −20°C until needed.

The bacterial 16S rRNA gene was amplified using the forward bacterial primer GM5F (5′-CCTACGGGAGGCAGCAG-3′) with a GC clamp (Muyzer et al., 1993) and the universal reverse primer D907R (5′-CCGTCAATTCCTTTRAGTTT-3′) (Amann et al., 1992). Archaeal amplification was conducted using the forward primer Arc333 with GC clamp and the reverse primer Arc958R (Struchtemeyer et al., 2005). Thermal cycling of all PCR reactions was performed with a “touchdown” PCR method (Muyzer et al., 1993). Denaturing gradient gel electrophoresis (DGGE) was then performed using the PCR-amplified bacterial and archaeal 16S rRNA gene products. The protocol described by Muyzer et al. (1993) was modified by using a 6% polyacrylamide gel and a 40–80% denaturing gradient, and running the gel in 1X Tris-acetate-EDTA (TAE) buffer solution for 16 h at 65 V at a constant 60°C temperature (Muyzer et al., 1993). The DGGE gels were stained with SYBR Safe and photographed under UV light. Bands of interest were excised and suspended in 30 μl of PCR-grade water; 5 μl of the excised DNA solution was used as template DNA for PCR reactions as described above. The 16S rRNA gene PCR products amplified from the excised DGGE bands were then sequenced at the Oklahoma Medical Research Foundation (Oklahoma City, OK) on an ABI 3730 capillary sequencer using the primers mentioned above.

Bacterial 16S rRNA gene clone libraries were prepared from DNA extracted using the PowerSoil DNA Isolation Kit from thiosulfate-using and methanogenic enrichments that had been transferred at least four times. Fragments of nearly full length 16S rRNA gene were generated by PCR amplification using the primers 8F and 1492R (Stackebrandt and Goodfellow, 1991) and cloned into *Escherichia coli* using the TOPO TA Cloning Kit for Sequencing (Invitrogen Corp., Carlsbad, CA). Transformed colonies were picked and transferred to 96-well microtiter plates containing Luria-Bertani broth with 25% (v/v) glycerol and 50 μ g·ml^−1^ kanamycin, and grown overnight at 37°C. Plasmid DNA was purified from the transformed cells and sequenced using the M13F and M13R vector flanking regions as priming sites on an ABI 3730 capillary sequencer (Microgen: Laboratory for Genomics and Bioinformatics, Oklahoma City, OK).

The consensus bacterial 16S rRNA gene sequences were obtained using Sequencher version 5.1 (Gene Codes, Ann Arbor, MI). The software package mothur v.1.30.2 (Schloss et al., 2009) was used to remove vector and chimera sequences, and define operational taxonomic units (OTUs) at a 97% identity level. A sequence representing each OTU was chosen and compared to previously reported 16S rRNA gene sequences using the BLASTN version 2.2.21+ (Zhang et al., 2000). A naïve Bayesian rRNA classifier (Wang et al., 2007) based on a 80% confidence threshold available through the RDP was used to assign phylogenetically consistent taxonomy to each representative sequence. Each representative sequence was rescreened to exclude chimeras using Pintail (Ashelford et al., 2005). Sequences that most closely matched the representative OTU sequence and selected outgroup sequences were aligned with CLUSTALX (version 1.81) (Thompson et al., 1997). Tree construction was performed using the Neighbor-Joining method (Saitou and Nei, 1987) with bootstrap values of 70% or greater used to determine each clade based on 1000 replicates (Felsenstein, 1985). Each of the four bacterial 16S rRNA gene sequence libraries contained between 16 and 21 sequences, for a total of 74 near full-length sequences (approximately 1480 bp).

### Accession numbers

The archaeal 16S rRNA gene sequences were deposited in GenBank under Accession numbers JQ014192-JQ014196. The bacterial sequences were deposited under Accession No. JQ014197-JQ014201 and KF137640.

## Results

### Cell enumerations and sulfide production

MPN tubes with sulfite and thiosulfate had sulfide concentrations above control values in at least two tubes at all dilutions (up to 10^−6^) regardless of whether VFAs were present or not present (Tables S1, S2). With sulfate as the electron acceptor, positive MPN tubes were detected at dilutions up to 10^−5^. The MPN of the thiosulfate-reducing microorganisms was estimated at greater than 1.1 × 10^6^ cells·ml^−1^ for both media with and without VFAs (Table 1). The MPN of sulfide producers using other sulfidogenic electron acceptors was lower than that for thiosulfate reducers (Table 1). The MPN of sulfite-reducing microorganisms was 2.2 × 10^5^ cells·ml^−1^ for both media with and without the VFA amendments. Sulfate-reducing microorganisms were only detected in medium with VFAs and had a MPN of 2.4 × 10^4^ cells·ml^−1^ (Table 1). The MPN of polysulfide-reducing microorganisms was 4.6 × 10^5^ cells·ml^−1^ and 4.3 × 10^4^ cells·ml^−1^, in medium with and without VFAs, respectively. The MPN data indicated that thiosulfate reducers were the most numerous cultured, sulfide producers in the PIG scrapings from this facility.

**Table 1:** **Most-probable numbers of sulfide producers in media with and without volatile fatty acids (VFA) and with different electron acceptors**.

**Electron acceptor**	**VFA presence**	**MPN (cell • ml^−1^)**	**Two-sided 95% confidence interval (cell • ml^−1^)**	
			**Lower**	**Upper**
Sulfate	+	2.4 × 10^4^	5.8 × 10^3^	9.9 × 10^4^
	−	BDL^a^	BDL	BDL
Sulfite	+	2.2 × 10^5^	1.0 × 10^5^	4.5 × 10^5^
	−	2.2 × 10^5^	1.0 × 10^5^	4.5 × 10^5^
Thiosulfate	+	>1.1 × 10^6^	NA^b^	NA
	−	>1.1 × 10^6^	NA	NA
Polysulfide	+	4.6 × 10^5^	2.2 × 10^5^	9.7 × 10^5^
	−	4.3 × 10^4^	1.6 × 10^4^	1.1 × 10^5^

a BDL (below detection limit; <100 cells • ml^−1^) indicates none of the tubes were scored as positive.

b NA, not applicable because all MPN tubes were positive for sulfide production with thiosulfate as electron acceptor.

MPN tubes with thisosulfate, both with and without the VFAs, produced large amounts of sulfate, up to 7.7± 1.8 to 9.4 ± 0.64 mM in the 10^−4^ to 10^−6^ dilutions (Tables S1, S2). Sulfate accumulation in MPN tubes with thiosulfate far exceeded that in controls that lacked an added electron acceptor. The production of both sulfide and sulfate in the thiosulfate-using MPNs provided evidence for the disproportionation of thiosulfate (Jørgensen, 1990), but this activity was lost upon subsequent enrichment and cultivation attempts.

### Thiosulfate reduction and methanogenesis with yeast extract as the electron donor

Thiosulfate-reducing MPN cultures with and without VFAs and their respective controls (e.g., cultures amended with VFAs but not thiosulfate as well as those lacking both thiosulfate and VFAs), were transferred into media of the same composition. The total amount of thiosulfate used by enrichments transferred four times was relatively low, 7.9 ± 3.4 and 15.7 ± 2.8 μmoles in enrichments with and without VFAs, respectively. Similarly, only small amounts of sulfide were produced, 17.8 ± 2.1 and 11.2 ± 5.9 in enrichments with and without thiosulfate, respectively. The low levels of thiosulfate use and sulfide production suggested that the enrichments were electron donor deficient. To gain a better understanding of carbon cycling in these enrichments, VFA use and methane production were measured at the end of a 4-week incubation period (Table 2). Little to no change in the total amount of butyrate (≤0.2 ± 0.02 μmoles) and valerate (≤0.3 ± 0.03 μmoles) were noted. Enrichments with VFAs and thiosulfate, with thiosulfate and without VFAs, and without either VFAs or thiosulfate produced substantial amounts of acetate, 12.1 ± 2.2, 8.1 ± 4.8, and 9.0 ± 3.0 μmoles, respectively (Table 2). All of the enrichments produced between 3 and 4 μmoles of propionate. In addition, low levels of isobutyrate, 2-methylbutyrate, and isovalerate were detected in our assays. The production of branch-chained fatty acids suggests the syntrophic cooperation in methanogenic degradation of amino acids. Total VFA levels remained unchanged in uninoculated controls (data not shown). Large amounts of methane were produced in enrichments that lacked thiosulfate addition (Table 2). The production of acetate, propionate, and branched-chain fatty acids suggested that the small amount of yeast extract present in the medium was being utilized as an electron donor to support thiosulfate reduction or ultimately methane production. Yeast extract likely mimics the organic compounds exchanged by members of the anaerobic community. To test this possibility, the amount of yeast extract in the medium was varied. Figures S1A,B shows that thiosulfate reduction and the concomitant production of sulfide was dependent on the amount of yeast extract amended to the cultures. The amount of methane produced was also proportional to the amount of yeast extract present (Figure S1C). Very little methane was produced in the absence of yeast extract. The data from Figure S1 confirmed that yeast extract was an electron donor to support biological thiosulfate reduction and methane production in the enrichment cultures.

**Table 2:** **Net production of methane and volatile fatty acids by enrichments transferred four times in medium (containing 0.05% yeast extract) with and without thiosulfate and volatile fatty acids**.

**Additions**	**Methane (μmoles)^a^**	**Acetate (μmoles)**	**Propionate (μmoles)**	**Butyrate (μmoles)**	**Isobutyrate (μmoles)**	**2-methyl-butyrate (μmoles)**	**Valerate (μmoles)**	**Isovalerate (μmoles)**
Thiosulfate + VFA	0.11 ± 0.02^a^	12.1 ± 2.25	3.5 ± 0.5	0.1 ± 0.3	0.6 ± 0.1	0.9 ± 0.13	0.1 ± 0.4	1.2 ± 0.2
Thiosulfate	0.09 ± 0.04	8.1 ± 4.8	3.0 ± 1.7	0.2 ± 0.1	0.5 ± 0.3	0.9 ± 0.1	BDL^b^	1.1 ± 0.2
VFA	14.6 ± 1.6	9.0 ± 3.0	4.1 ± 1.3	0.2 ± 0.3	0.6 ± 0.2	0.6 ± 0.3	0.3 ± 0.03	0.9 ± 0.5
None	26.60 ± 3.81	2.0 ± 2.6	4.0 ± 1.2	BDL	0.7 ± 0.2	0.8 ± 0.1	BDL	1.16 ± 0.2

a Values for methane and VFAs corrected for initial levels at the time of inoculation.

b BDL (Below Detection Limit) indicates a concentration less than 0.05 mM, the detection limit of the gas chromatograph.

### Molecular analyses of thiosulfate-reducing and methanogenic enrichments

**Figure 1:**
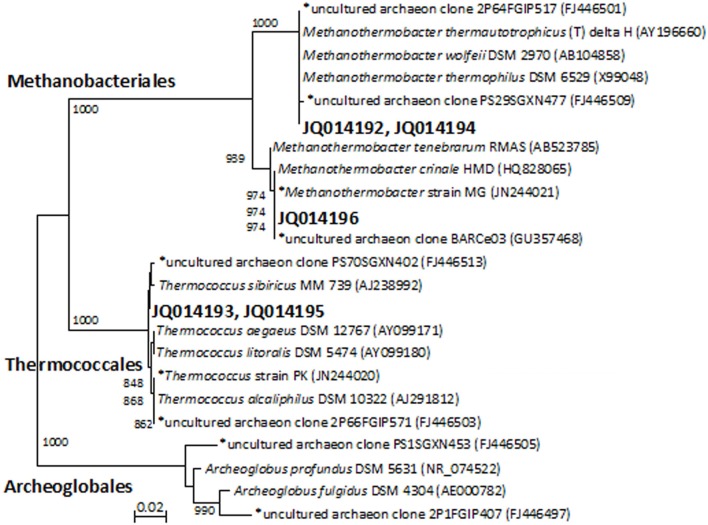
**Phylogenet ic relationships of archaeal 16S rRNA gene sequences obtained from the excised DGGE bands from enrichments and MPN cultures**. The tree is constructed from approximately 350 bp 16S rRNA gene sequences using the neighbor-joining algorithm. One thousand bootstrap replications were performed; only values greater than 700 are shown. The accession numbers shown in bold are of representative sequences from the current study and sequences beginning with an ^*^ are from samples, enrichments, or isolates taken from the same field in our previous studies (Duncan et al., 2009; Gieg et al., 2010; Stevenson et al., 2011; Davidova et al., 2012). Bar: 0.02 nucleotide substitutions per nucleotide.

Denaturing gradient gel electrophoresis profiles for archaea were generated from the initial MPN cultures and enrichments that had been transferred two and four times (Figure S2, showing the fourth transfer). The intensity of the archaeal DGGE bands detected in thiosulfate-reducing cultures decreased with increasing transfers. By the fourth transfer, archaeal sequences were not detected in thiosulfate-reducing enrichments. Denaturing gradient gel electrophoresis band sequences obtained from MPN (JQ014192) and second transfer enrichments (JQ014194) were both closely affiliated with *Methanothermobacter thermautotrophicus* type-strain Delta H (Figure 1). By the fourth transfer, one dominant archaeal band was evident in enrichments with and without a VFA amendment that lacked a sulfidogenic electron acceptor (Figure S2). The sequence (JQ014196) was classified to the genus *Methanothermobacter* with 100% confidence and shared 100% sequence identity to the sequence of *Methanothermobacter crinale* strain HMD (GenBank Accession No. HQ828065) (Figure 1). The presence of the *Methanothermobacter* related sequence is consistent with the methane production from metabolism of yeast extract in the absence of a sulfidogenic electron acceptor. A second DGGE band was sequenced from the initial MPN cultures (JQ014193) and after the second transfer (JQ014195). These two sequences fell into the same 97% OTU and classified in the genus *Thermococcus* at 100% confidence. The most similar isolate 16S rRNA sequence to JQ014193 and JQ014195 is that of the type strain of *Thermococcus sibiricus* MM739 (AJ238992) (Figure 1).

**Figure 2:**
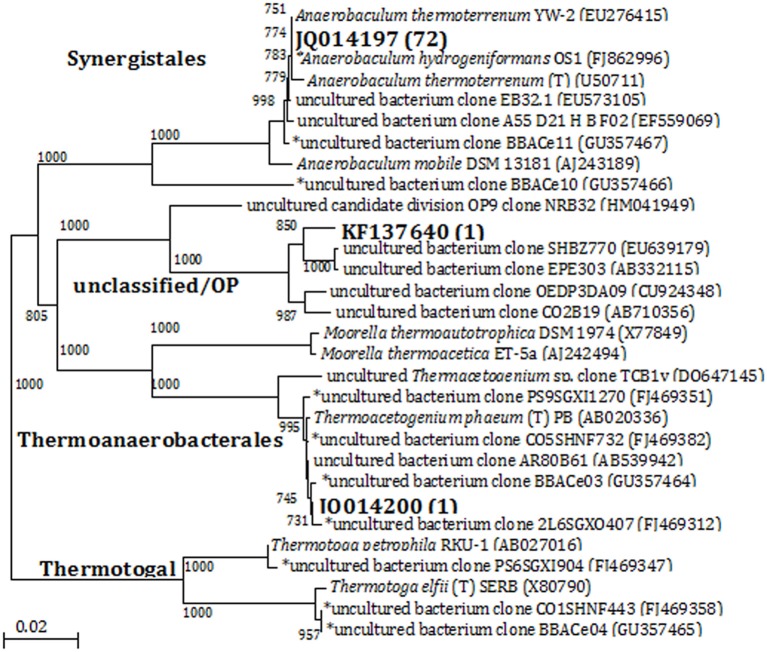
**Phylogenetic relationships of bacterial 16S rRNA gene sequences obtained from enrichments by the 4th transfer**. The tree is constructed from approximately 1300 bp 16S rRNA gene sequences using the neighbor-joining algorithm. One thousand bootstrap replications were performed; only values greater than 700 are shown. The accession numbers shown in bold are of representative sequences from the current study; the number in parentheses following bold accession numbers is the total number of sequences obtained for this OTU (97% similarity). Sequences beginning with an ^*^ are from samples, enrichments, or isolates taken from the same field in our previous studies (Duncan et al., 2009; Gieg et al., 2010; Stevenson et al., 2011; Davidova et al., 2012). Bar: 0.02 nucleotide substitutions per nucleotide.

Denaturing gradient gel electrophoresis analysis indicated that cultures with thiosulfate had relatively high bacterial diversity based on the number of bands in the profiles, which decreased with increasing transfers (Figure S3, showing the fourth transfer). A group of highly visible and similar bands were seen in all of the enrichments with each transfer, indicating the presence of a small but dominant group of bacteria present in these cultures regardless of electron donor or acceptor conditions of the medium. Although the bands showed high resolution and intensity, the physical separation between them was too small to allow for accurate excision and direct sequencing. In this case, bacterial 16S rRNA gene sequence libraries (total number of sequences: 74) were generated from the enrichments that had been transferred four times. Three OTUs (at 97% similarity) were obtained from the four enrichment conditions. Phylogenetic analysis separated the sequences into three clades at the order level: *Synergistales, Thermoanaerobacterales, and Unclassified/OP9* (Figure 2). Of the 74 sequences from these enrichments, 72 of them were classified into the same 97% OTU (JQ014197) within the phylum *Synergistetes* affiliated with the genus *Anaerobaculum* at 100% sequence identity. The *Anaerobaculum*-related sequences formed the majority of clones obtained from each of the four enrichment conditions. The presence of a common bacterial sequence in all four enrichment conditions is consistent with the DGGE analysis, which detected some closely adjacent bands at the bottom of each lane (Figure S3), beginning with the MPN dilutions (data not shown). Enrichments with thiosulfate and lacking VFAs had one OTU (JQ014200) that grouped within the phylum *Firmicutes* in the family *Thermoanaerobacteraceae* at 100% confidence (Figure 2). This OTU (JQ014200) showed a 99.6% sequence identity to the cultured isolate *Thermacetogenium phaeum* PBT (GenBank Accession No. AB020336). A third OTU (KF137640), from enrichments without thiosulfate but containing VFAs showed highest similarity to unclassified uncultured clones and those identified as affiliated with Candidate Division OP9.

### Iron corrosion by the thiosulfate-reducing enrichments

In petroleum systems, complex forms of organic matter analogous to yeast extract are ubiquitous in oil formations and production facilities. Such organic matter likely originates from the maturation of kerogen, the degradation of complex hydrocarbons, extracellular polymeric substances in biofilm matrices, cellular material and combinations thereof (Duncan et al., 2009). In order to simulate water-soluble organic matter in oil field systems, yeast extract concentration was used as the electron donor to evaluate biocorrosion of carbon steel by the thiosulfate-reducing enrichment, which produces sulfide and organic acids, and the methane-producing enrichment, which produces organic acids but not sulfide.

**Figure 3:**
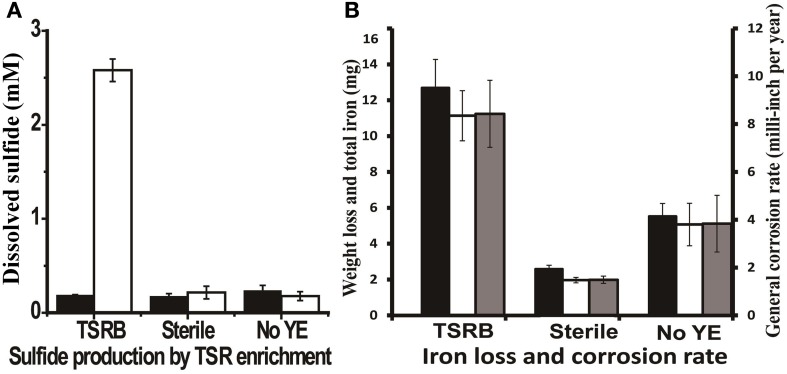
**Sulfide production and corrosive activities of thiosulfate-reducing enrichments. (A)** Dissolved sulfide accumulation in the medium after 14 days incubation. The black bars represent the dissolved sulfide at zero time, and the clear bars represent dissolved sulfide at the end point of experiment. **(B)** Weight loss and total iron released from coupon in the cultures after 14 days incubation. The black bars represent corrosion determined by total iron analysis; the clear bars indicate corrosion as determined by weight loss; and the gray bars are the general corrosion rates calculated based on the weight loss per year. The medium contained 1 g/l yeast extract. Abbreviations: TSRB, thiosulfate-reducing enrichment; Sterile, controls with autoclaved inocula; and No YE, inoculated controls without YE.

Thiosulfate-reducing enrichments with carbon steel coupons turned the medium completely black after 3 days, most likely due to the precipitation of iron sulfides. The accumulation of up to 2.5 ± 0.12 mM dissolved sulfide was detected over a 14-day incubation period in the presence of coupons (Figure 3A), which was comparable to the amount of sulfide produced by the same enrichment without coupons (*P* = 0.046). Meanwhile, very thick, fluffy corrosion products formed on the surfaces of the coupons and much of the deposits flaked off into the medium. Figure S4A shows the FE-SEM image of a coupon immersed in the medium after 14 days. The rod-shaped cells, morphologically similar to *Anaerobaculum* species, can be seen underneath the precipitates. As can be seen in Figure 3B, the general corrosion rate as determined by weight loss (8.43 ± 1.40 mpy) is much higher than that in autoclaved (1.49 ± 0.15 mpy, *P* = 0.003) and substrate-unamended controls (3.83 ± 1.18 mpy, *P* = 0.002). The total iron analysis followed the same trend, indicating that total iron analysis can be used as a complementary approach to assess general corrosion. Meanwhile, high-resolution, three-dimensional profilometric scanning images demonstrated that the damage caused on the surface of the coupon in the active culture is more pronounced than in the autoclaved controls (Figures S5A,B). Unlike the relatively smooth steel surface in the control, severe pitting was evident on the coupon surface in the presence of active thiosulfate-reducing bacteria. The pit counting analysis showed 59 pits with sizes of more than 10 μm in diameter and 20 μm in depth, whereas no pits of this size was detected in the sterile control. Twenty-nine pits in coupons exposed to active thiosulfate-reducing bacteria were greater than 20 μm in diameter and depth.

### Biocorrosion by methanogenic enrichment cultures

**Figure 4:**
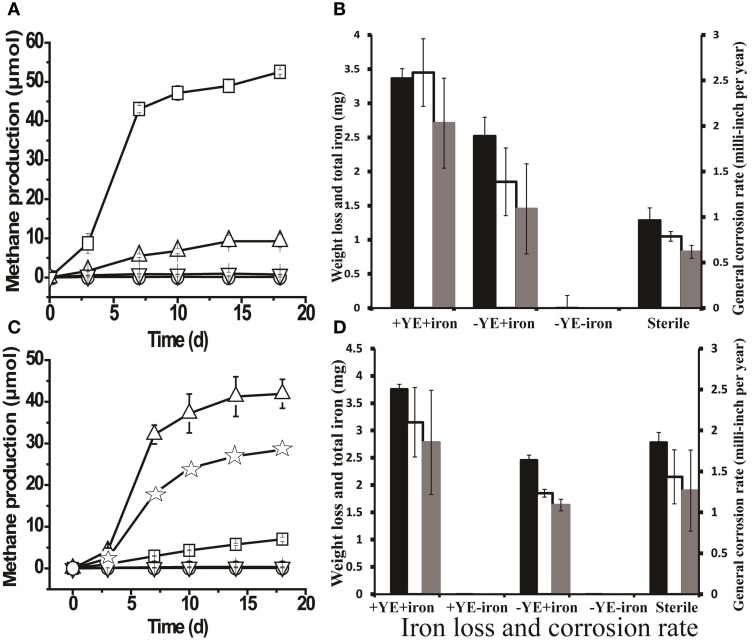
**Methane production and corrosive activities of the methanogenic enrichment**. **(A)** Methane production by methanogenic cultures grown with yeast extract under various conditions: □, active inocula with yeast extract (YE) and a carbon-steel coupon in the medium; ∆, active inocula with only the coupon in the medium; ∇, active inocula without YE or the coupon; and ○, autoclaved inocula with YE and coupon. **(B)** Weight loss and total iron released from the coupon in methanogenic enrichments previously grown with YE only. **(C)** Methane production by methanogenic cultures after six successive transfers with iron as the sole source of electrons in the medium: ∆, active inocula with YE and a coupon in the medium; ☆, active inocula with only YE in the medium; □, active inocula with only a coupon in the medium; ∇ and ○ (overlapping data) refer to the negative controls, active inocula without YE or the coupon and autoclaved inocula with YE and a coupon, respectively. **(D)** Weight loss and total iron released from coupon in the cultures of methanogenic enrichment after 6 transfers with iron granules. The black bars in Figures 4B,D indicate the corrosion determined by total iron analysis; the clear bars in Figures 4B,D represent the corrosion determined by weight loss; and the gray bars in Figures 4B,D are general corrosion rate calculated based on the weight loss data. +YE+iron: with both YE and coupon in the medium; Sterile: autoclaved inocula with both YE and coupon in the medium;−YE+iron: with only the coupon in the medium;−YE−iron: without YE or the coupon in the medium; +YE−iron: with only YE in the medium. The methane production (μ mols) was calculated based on a 15-ml headspace.

The highest amount of methane (52.52 ± 0.56 μmols) was produced when methanogenic enrichments were grown a medium containing yeast extract and a coupon (Figure 4A). Meanwhile, only negligible methane production (1.68 ± 0.19 μmols) was detected in controls without yeast extract or a coupon. The FE-SEM confirmed biofilm formation by the methanogenic enrichment (Figure S4B, the arrows indicate the cells inside the biofilm matrix). In Figure 4B, it can be seen that the methanogenic enrichment resulted in significantly more iron loss than in the autoclaved control (*P* = 0.04). However, the general corrosion rate (2.03 ± 0.49 mpy) caused by the methanogenic enrichment was approximately 4 times less than thiosulfate-reducing enrichment (8.43 ± 1.40 mpy, *P* = 0.002). Surface profilometry analysis revealed that the methanogenic enrichment created more pitting corrosion to the coupon surface (Figures S5C,D) relative to the sterile control, but far fewer pits were observed (6) than the thiosulfate-reducing enrichment (59).

As seen in Figure 4A, 9.27 ± 1.68 μmoles of methane were produced after 18 days of incubation in a medium that lacked yeast extract but did have a coupon. Intriguingly, the corrosion noted in the absence of yeast extract was slightly higher than that in the autoclaved control (Figure 4B). The above observations prompted us to investigate corrosion by the methanogenic enrichment with iron as the sole source of electrons. Methane production was observed when the methanogenic enrichment was repetitively transferred in a medium with iron granules as the sole source of electrons. After six successive transfers, the corrosive activity of the methanogenic enrichment was quantified. The methanogenic enrichment produced methane with the coupon alone, but more methane was produced when yeast extract was also available in the medium (Figure 4C). This suggests that the fermentative organisms in the consortium survived the repeated transfers. Cell morphologies similar to *Anaerobaculum* species (the large rod indicated by the bottom arrow in Figure S4D) and *Methanothermobacter* species (the small rod indicated by the upper arrow in Figure S4D) (Cheng et al., 2011) were both observed on the coupon surface. However, cells attached to the surface were morphologically most similar to *Methanothermobacter* (Cheng et al., 2011) when the iron served as the sole source of electrons. Contrary to the previous observation with the methanogenic enrichment grown with yeast extract (Figure 4B), the corrosion caused by methanogenic enrichment, which was repetitively transferred with only iron granules, was not significantly different (*P* = 0.243) than that observed with the autoclaved control (Figure 4D). The corrosion observed in Figure 4B could be explained by carry-over of yeast extract or other nutrients with the inoculum. Similar to the methanogenic enrichment grown on yeast extract, more corrosion was observed when the methanogenic enrichment maintained in medium with iron granules was grown in a medium with yeast extract than with the coupon alone (Figure 4D), confirming that the fermentative organisms in the methanogenic enrichment still survived after six transfers with iron, and that they enhanced corrosion when yeast extract was available.

## Discussion

MPN studies of oil pipeline PIG samples confirmed that microorganisms that use thiosulfate, sulfite, or polysulfide as electron acceptors were numerically more dominant than those that use sulfate as an electron acceptor (Table 1). Thiosulfate-users were at least 100-fold more numerous than sulfate-reducing bacteria. Our findings are consistent with studies of corrosion at a Canadian oil facility where about 30% of the isolated sulfidogenic organisms were strict sulfite reducers, 50% used only thiosulfate and elemental sulfur as electron acceptors, and only 20% were strict sulfate-reducing bacteria (Obuekwe et al., 1983; Obuekwe and Westlake, 1987). Nielsen (1991) also showed that biofilm formation and hydrogen sulfide production increased by a factor of about two in enrichments supplemented with either sulfite or thiosulfate as the electron acceptor compared to sulfate-reducing enrichments (Nielsen, 1991). A more recent study of an Indian oil field found that MPN of thiosulfate-reducing bacteria were 4- to 8-fold higher than sulfate-reducing bacteria (Agrawal et al., 2010). Although the number of sulfate-reducing bacteria found in the Indian oil field sites and the ANS were similar (10^5^ cells·ml^−1^), the MPN of thiosulfate-reducing bacteria was higher in our study. The overall conclusion from the above analysis reinforces the importance of non-sulfate-reducing, sulfidogenic microorganisms and the utilization of sulfur oxyanions other than sulfate in the biocorrosion of petroleum facilities. We detected evidence of thiosulfate disproportionation in our MPN cultures inoculated with PIG fluids. Disproportionation of inorganic sulfur molecules has not been widely studied in oil production facilities and may have significant implications for the biogeochemical cycling of sulfur in many environments (Jørgensen, 1990; Park et al., 2011). Although thiosulfate disproportionation activity was lost upon transfer, microorganisms with this metabolic capacity should also be acknowledged as potential contributors to pipeline biocorrosion problems.

Molecular analysis was employed to link the physiochemical properties of the enrichment cultures to specific microbial interactions. Sequences related to *Anaerobaculum* sp. were identified in all four of the enrichments after four transfers. This finding is consistent with the detection by DGGE analysis of a common band in all of the enrichments. Members of the genus *Anaerobaculum* belong to the family *Synergistaceae* and are fermentative organisms that produce acetate, CO_2_, and H_2_ as their major metabolic end products (Menes and Muxí, 2002; Maune and Tanner, 2012). *Anaerobaculum* sp. utilize a wide range of organic substrates including glucose, various organic acids, peptides, and complex organic substrates such as yeast extract (Menes and Muxí, 2002; Maune and Tanner, 2012). *Anaerobaculum* sp. are also capable of reducing thiosulfate, elemental sulfur, and cystine, but not sulfate or sulfite, to hydrogen sulfide (Rees et al., 1997; Menes and Muxí, 2002; Maune and Tanner, 2012). The bacterial clone libraries, due to the number of sequences examined, identified only dominant members of the enrichments and were not intended to fully sample the diversity. In addition, a number of other thermophilic, fermentative, thiosulfate-reducing bacteria belonging to the genera *Thermotoga* (Fardeau et al., 1997), *Thermoanaerobacter* (Fardeau et al., 2000), and *Garciella* (Miranda-Tello et al., 2003) have been isolated from high temperature petroleum reservoirs and processing facilities worldwide. We also detected sequences related to *Thermoanaerobacter* sp. in some of our enrichments. The cultivation-dependent and cultivation-independent approaches employed in this work and by others (Fardeau et al., 1997; Magot et al., 1997; Fardeau et al., 2000; Orphan et al., 2000; Miranda-Tello et al., 2003; Duncan et al., 2009; Stevenson et al., 2011) collectively show the ubiquity and the importance of fermentative, thiosulfate-reducing bacteria within oil fields. However, a large majority of studies related to corrosion have traditionally focused on sulfate-reducing bacteria and their accepted role as the primary sulfidogenic organisms in petroleum environments. The potential of thermophilic, fermentative sulfidogenic microorganisms to catalyze biocorrosion processes has been underappreciated. In this regard, it is imperative to evaluate the role of these organisms in biocorrosion of carbon steel in order to provide further guidance for better monitoring and preventing corrosion in the energy sector.

Anaerobic corrosion of iron leads to the mass loss of the iron surface and the release of ferrous iron into the aqueous phase (De Windt et al., 2003). The measurement of iron mass loss is a long established routine method and thus has been most frequently employed to determine corrosion (Enning et al., 2012). Although few studies are available (De Windt et al., 2003) attempting to measure the total iron dissolved from metal surfaces, we also performed total iron analysis to assess the corrosive potential of thermophilic, thiosulfate-reducing bacterial enrichments. We generally found that such procedures can be used as a good approach to quantify corrosion. The data clearly show that the corrosion caused by communities containing thiosulfate-reducing bacteria was more pronounced than the abiotic controls. The higher number of pits formed by thiosulfate-reducing bacteria show that they cause severe biocorrosion and indicate that these organisms are involved in pitting corrosion. It has been suggested that the accumulation of VFAs, particularly acetate (Callbeck et al., 2013), and hydrogen sulfide changes the microenvironment at the metal-liquid surface and initiates pitting corrosion (Campaignolle et al., 1996; Miranda et al., 2006). However, it was unclear whether our thiosulfate-reducing bacteria enrichment caused corrosion by the above mechanism. By using electrochemical measurements, Magot et al. demonstrated that mesophilic thiosulfate-reducing bacteria isolated from a corroding offshore Congo oil well induced severe pitting (Magot et al., 1997), with corrosion penetration rates of up to 4 mm per year. They concluded that the fermentative organisms were the causative agents for the failure of pipelines in Elf Congo, which experienced severe bacterial pitting corrosion within a year after replacement. Another study revealed that the dominant group of thiosulfate-reducing bacteria were *Enterobacteriaceae*, which was responsible for severe localized corrosion of carbon steel in a marine system (Bermont-Bouis, 2007). It should be noted that thiosulfate reduction is a common feature of most sulfate-reducing bacteria, and these organisms were implicated as the principal cause of biocorrosion in the presence of thiosulfate (Miranda et al., 2006).

An archaeal co-culture of *Thermococcus* sp. and *Methanothermobacter* sp. isolated from the ANS caused corrosion when incubated in a medium with both yeast extract and elemental iron (Davidova et al., 2012). The presence of a hydrogen-consuming methanogen increased production of VFAs by *Thermococcus* sp. compared to *Thermococcus* sp. growing alone. It is likely that the accumulation of VFAs was an important factor in corrosion. It is reasonable to expect that the *Anaerobaculum* sp. and *Methanothermobacter* sp. present in our enrichments act in a similar manner to impact biocorrosion of carbon steel. Indeed, we demonstrated that the methanogenic enrichment produced methane and a similar suite of VFAs as was found with the *Thermococcus* sp.-*Methanothermobacter* sp. coculture through the fermentation of complex organic matter such as yeast extract. The methanogenic enrichment with *Anaerobaculum* sp. and *Methanothermobacter* sp. formed a biofilm on the surface of the coupon and corroded the coupons, but to a lesser extent than the thiosulfate-reducing enrichment. Hydrogenotrophic methanogens are commonly isolated from petroleum formations and are known to make up a significant portion of the indigenous microbial communities in such systems (Cheng et al., 2011; Mayumi et al., 2011; Nakamura et al., 2012). Sequences related to *M. crinale* and *M. thermautotrophicus* have been identified in the ANS facility and other petroleum environments (Orphan et al., 2000; Pham et al., 2008; Duncan et al., 2009; Gieg et al., 2010). Clone library analysis of the same PIG sample showed that 75% of the detected archaeal OTUs were affiliated to *Methanothermobacter thermautotrophicus* (Stevenson et al., 2011). Hydrogenotrophic methanogens allow a complex anaerobic food chain to develop in petroleum environments by serving as a biological electron acceptor. By virtue of their ability to form syntrophic partnerships with fermentative microorganisms, hydrogenothrophic methanogens can accelerate corrosion probably by enhancing acid production by fermentative microorganisms.

Our work utilized both culture-dependent and culture-independent methods to implicate the importance of thiosulfate-reducing bacteria and methanogenic microorganisms in the biocorrosion of a hot petroleum production facility. A fermentative, thiosulfate-reducing enrichment was more corrosive than a methanogenic enrichment. The information gathered argues for the crucial role played by non-sulfate-reducing, sulfidogenic organisms and methanogens in biocorrosion of pipelines under thermophilic conditions. Thiosulfate-mediated corrosion would be predicted to be most severe pipe sections where oxygen intrusion leads to thiosulfate production by its reaction with sulfides. Thus, traditional approaches to detect and prevent microbially induced corrosion should be reconsidered to place a greater emphasis on the numerically dominant, fermentative thiosulfate-reducing microorganisms in sulfur cycling and biocorrosion in the energy production systems.

### Conflict of interest statement

The authors declare that the research was conducted in the absence of any commercial or financial relationships that could be construed as a potential conflict of interest.
