# Supplementary material for: Roles of thermophilic thiosulfate-reducing bacteria and methanogenic archaea in the biocorrosion of oil pipelines
Source: Front Microbiol. 2014 Mar 6;5:89. doi: 10.3389/fmicb.2014.00089 (PMC3944610; doi:10.3389/fmicb.2014.00089)

Table S1. Mean endpoint sulfide and sulfate concentrations in most probable numbers tubes with different electron acceptors and volatile fatty acids as the electron donor [a].

Dilution	Electron acceptor added							
	Sulfate			Sulfite		Thiosulfate		
	Sulfide (mM)	Sulfate (mM)	Growth	Sulfide (mM)	Growth	Sulfide (mM)	Sulfate (mM)	Growth
10^{-2}	3.42 ± 0.09	0.22 ± 0.01	+	3.69 ± 0.03	+	5.35 ± 0.18	3.90 ± 0.99	+
10^{-3}	3.03 ± 0.21	1.16 ± 0.01	+	3.72 ± 0.21	+	6.02 ± 0.00	2.99 ± 0.11	+
10^{-4}	2.59 ± 0.35	2.90 ± 0.76	+	3.30 ± 0.24	+	3.96 ± 1.06	7.67 ± 1.81	+
10^{-5}	1.54 ± 0.21	5.21 ± 0.81	-	2.24 ± 0.97	+	4.34 ± 0.61	7.74 ± 1.17	+
10^{-6}	ND[b]	ND	-	3.00 ± 0.94	+	3.39 ± 0.17	9.42 ± 0.64	+
Uninoculated	1.14 ± 0.00	5.78 ± 0.00	-	1.05 ± 0.00	-	0.89 ± 0.02	2.27 ± 0.00	-

[a] Sulfide concentrations were corrected for background sulfide concentrations due to the use of sodium sulfide as the reductant. Positive (+) growth score indicates that at least two of three tubes at that dilution had sulfide concentrations that were at least 20% higher than sulfide in the uninoculated control of the same condition.

[b]ND, not detected. When sulfate was the electron acceptor, none of the tubes at dilutions 10^{-5} or higher had sulfide concentrations that were 20% higher than sulfide concentrations in the uninoculated control.

Table S2. Mean endpoint sulfide and sulfate concentrations in most probable numbers tubes with different electron acceptors and without volatile fatty acids[a].

Dilution	Electron acceptor added							
	Sulfate			Sulfite		Thiosulfate		
	Sulfide (mM)	Sulfate (mM)	Growth	Sulfide (mM)	Growth	Sulfide (mM)	Sulfate (mM)	Growth
10^{-2}	2.40 ± 0.37	2.83 ± 0.11	-	3.12 ± 0.22	+	5.30 ± 0.18	3.74 ± 1.15	+
10^{-3}	2.50 ± 0.15	3.05 ± 0.55	-	3.02 ± 0.11	+	5.85 ± 0.46	4.87 ± 0.76	+
10^{-4}	2.10 ± 0.43	4.17 ± 0.72	-	3.43 ± 0.16	+	5.55 ± 0.35	5.63 ± 0.72	+
10^{-5}	1.40 ± 0.63	4.62 ± 1.26	-	2.02 ± 0.23	+	3.48 ± 0.55	8.06 ± 0.72	+
10^{-6}	ND[b]	ND	-	2.78 ± 0.81	+	3.23 ± 0.07	9.19 ± 0.11	+
Uninoculated	2.21 ± 0.02	6.23 ± 0.00	-	1.62 ± 0.00	-	1.55 ± 0.02	1.81 ± 0.00	-

[a] Sulfide concentrations were corrected for background sulfide concentrations due to the use of sodium sulfide as the reductant. Positive (+) growth score indicates that at least two of three tubes at that dilution had sulfide concentrations that were at least 20% higher than sulfide in the uninoculated control of the same condition.

[b]ND, not detected. When sulfate was the electron acceptor, none of the tubes at dilutions 10^{-5} or higher had sulfide concentrations that were 20% higher than sulfide concentrations in the uninoculated control.

Figure. S1 Sulfide production and thiosulfate reduction by thiosulfate-reducing enrichments and methane production by methanogenic enrichments with different concentrations of yeast extract. (A) Sulfide production by the thiosulfate-reducing enrichments; (B) Thiosulfate reduction by the thiosulfate-reducing enrichments; (C) Methane production by the methanogenic enrichment. The percent concentration of yeast extract (YE) added to the medium is shown next to each plot. The methane production (μmols) was calculated based on a 15-ml headspace.

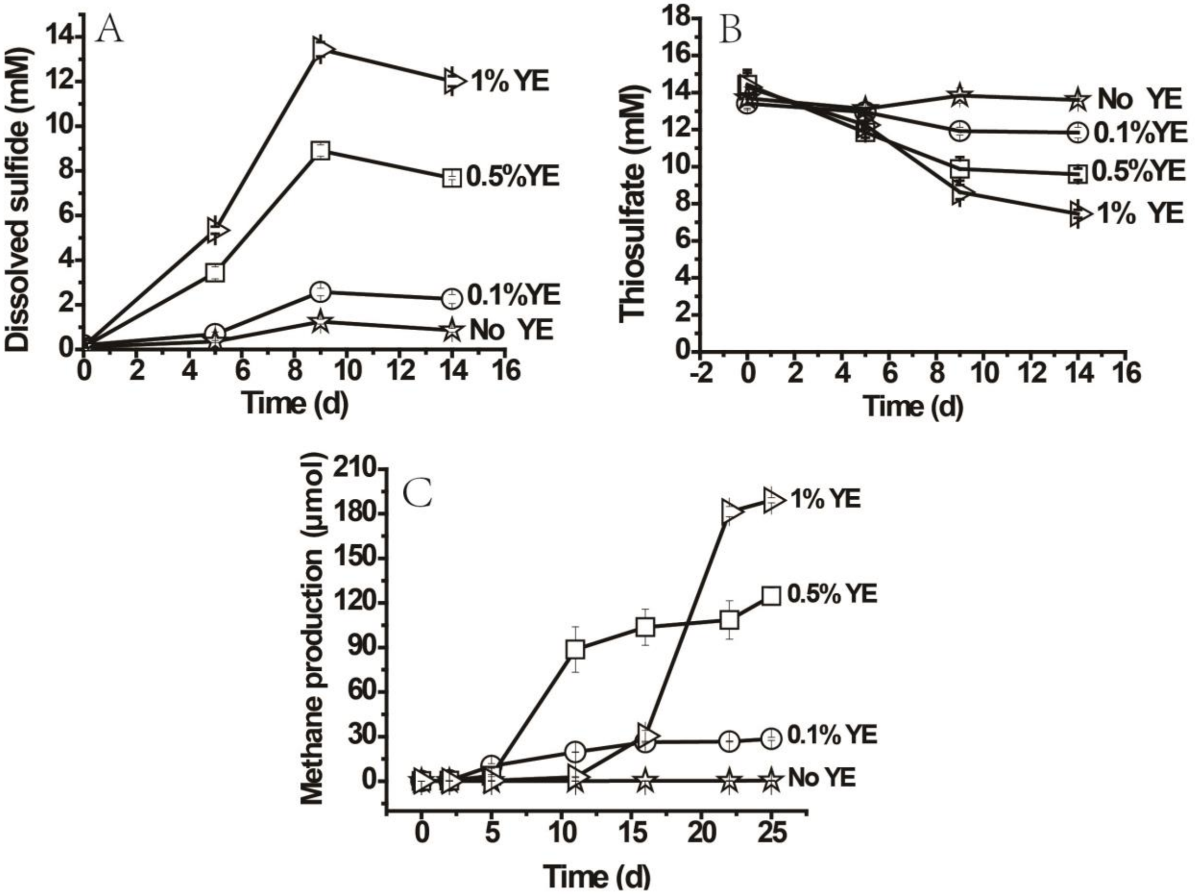

Figure.S2 Archaeal DGGE profile from enrichments transferred four times with or without VFAs and with or without thiosulfate added as electron acceptor. Lanes 1-5 represent to the following conditions: 1, *Methanocaldococcus jannaschii* DNA as positive control; 2, VFAs and Thiosulfate; 3, Thiosulfate and no VFAs; 4, VFAs without an electron acceptor; and 5, no VFAs and no electron acceptor.

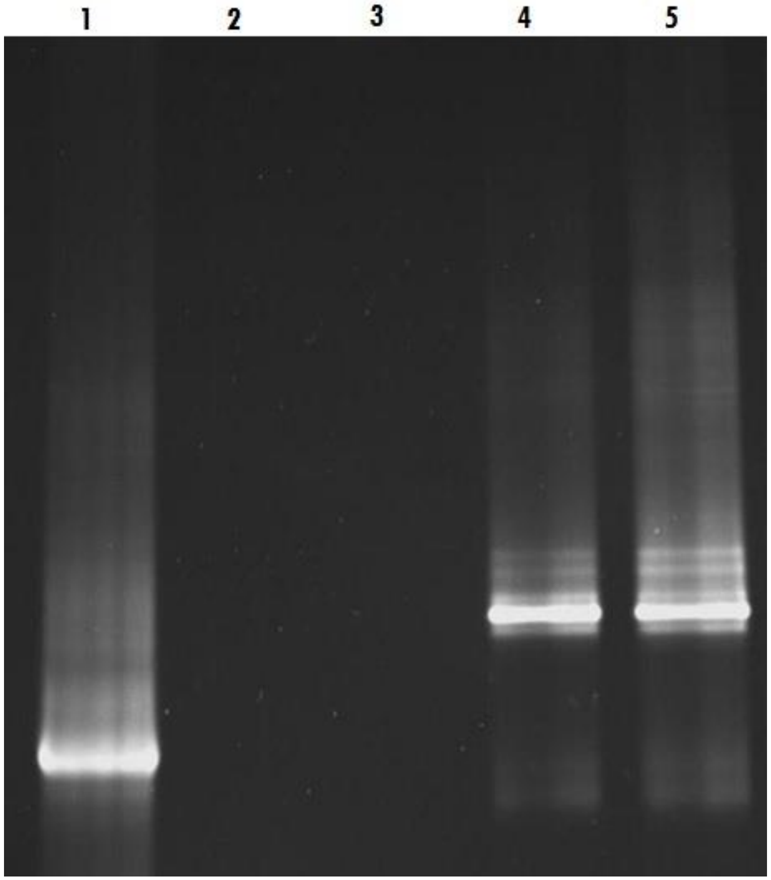

Figure. S3 Bacterial DGGE profile from enrichments transferred four times with or without VFAs and with or without thiosulfate added as electron acceptor. Lanes 1-5 represent to the following conditions: 1, *Bacillus licheniformis* as positive control; 2, VFAs+Thiosulfate; 3, No VFAs+thiosulfate; 4, VFAs+ no electron acceptors; 5, No VFAs+ no electron acceptors.

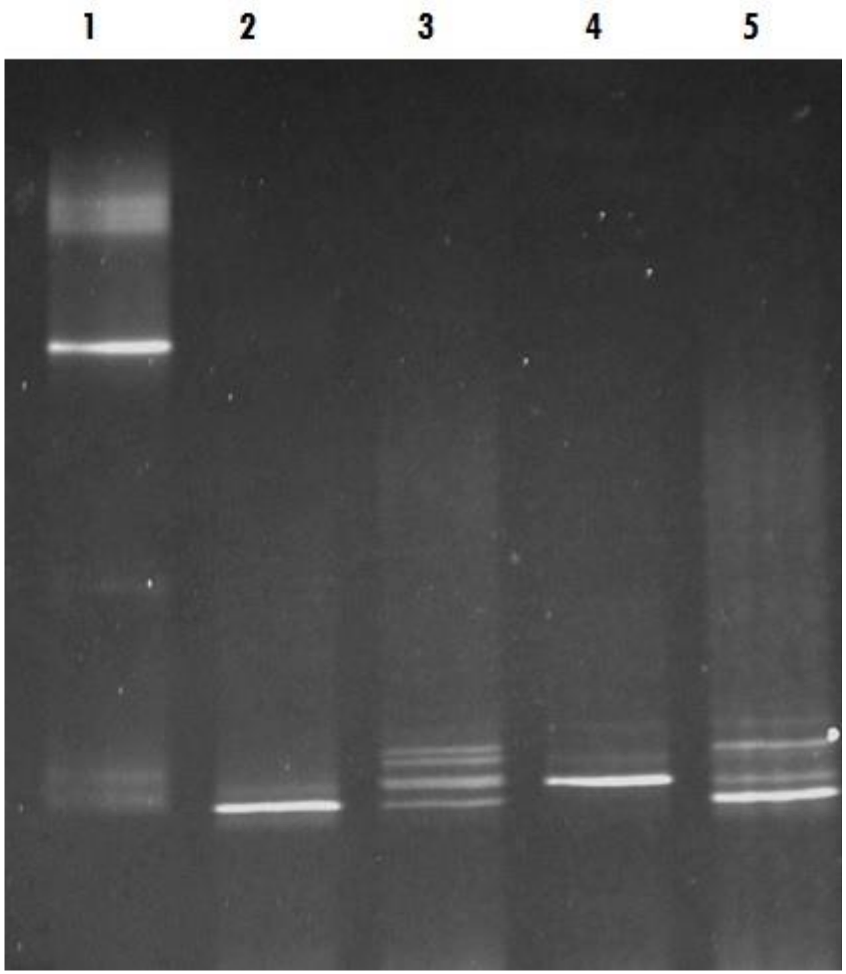

Figure.S4 Field-emission, scanning electron micrographs of corroding surfaces and cells colonization. (A) Cells of the thiosulfate-reducing enrichment embedded in the fluffy corrosion products; (B) Biofilm development by methanogenic enrichment grown in medium with yeast extract (YE); (C) Methanogenic enrichment grown with steel coupon as the sole source of electrons; (D) Methanogenic enrichment (after 6 successive transfers with iron granules) grown on YE. The rectangles in (A) and arrows in (B) and (D) indicate the cells attached on the surfaces. The scale bars next to the labels for (A), (B) and (C) are 2 µm whereas that for (D) is 1 µm.

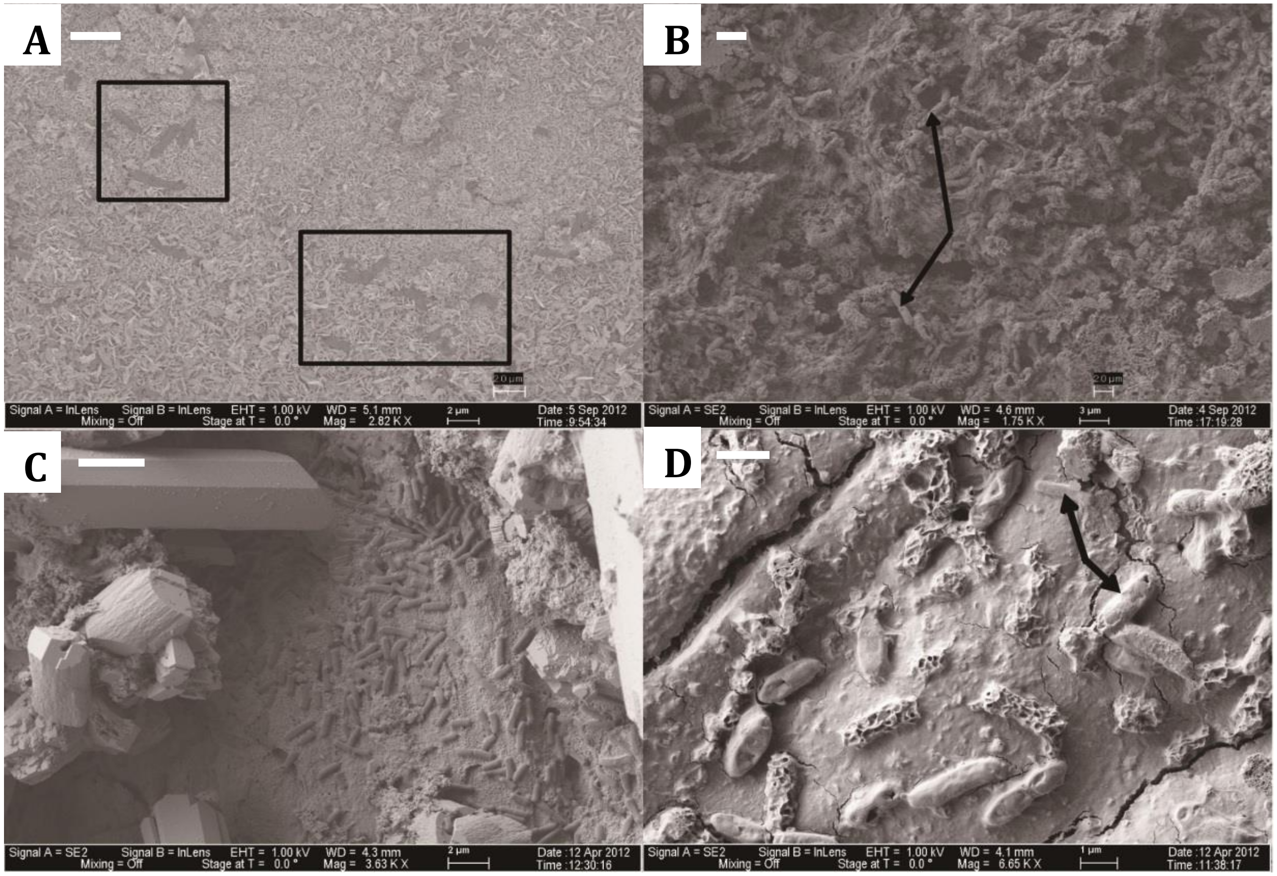

Figure. S5 Topological characterization of coupons by three-dimensional profilometry analysis. (A) Active inoculate of a thiosulfate-reducing enrichment; (B) Autoclaved thiosulfate-reducing enrichment inocula control; (C) Active methanogenic enrichment grown with yeast extract; (D) Autoclaved methanogenic enrichment control. The false colors correspond to the various depths of the measured points on the surface and the units for the scale bars are µm. The total surface area of the coupons is 71.16 mm^2 with a diameter of 9.53 mm.

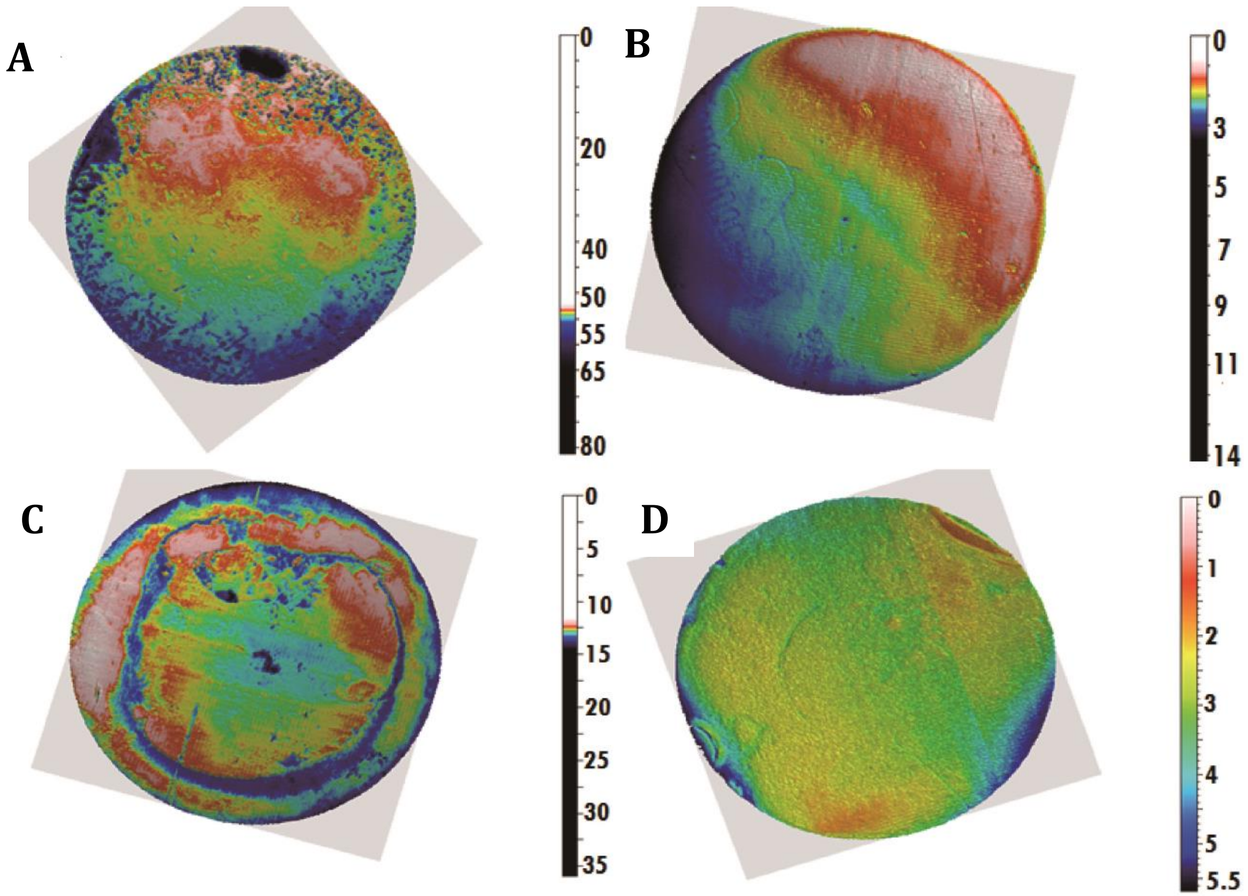

Supplement: Supplementary file 1 [file DataSheet1.PDF]
